# Clinical impact of early postoperative routine radiology in isolated pelvic injuries: a retrospective study

**DOI:** 10.1007/s00068-026-03243-z

**Published:** 2026-06-15

**Authors:** Eftychios Bolierakis, Paraskevas Ioannou, Sebastian L. Schubert, Rald V. M. Groven, Felix M. Bläsius, Maike Bode, Hatem Alabdulrahman, Frank Hildebrand, Till Berk

**Affiliations:** 1 Department of Orthopedics, Trauma, and Reconstructive Surgery, University Hospital RWTH Aachen, Pauwelsstrasse 30, 52074 Aachen, Germany; 2 Department of Diagnostic and Interventional Radiology, University Hospital RWTH Aachen, Pauwelsstrasse 30, 52074 Aachen, Germany

**Keywords:** Pelvic fractures, Postoperative imaging, Surgical fixation, Revision surgery, Treatment modification, Routine radiography

## Abstract

**Purpose:**

The clinical value of routine immediate postoperative conventional radiography following surgical fixation of pelvic fractures remains debated. This study assessed the impact of early postoperative X-rays on management decisions and revision surgery in patients with isolated pelvic ring or acetabular fractures.

**Methods:**

We conducted a retrospective cohort study at a Level 1 trauma center, including adults undergoing surgical treatment for isolated pelvic ring or acetabular fractures between 2020 and 2023. All patients received high-quality intraoperative fluoroscopy and postoperative conventional radiographs. The primary outcome was any change in standard postoperative care based on radiographic findings. The secondary outcome was revision surgery prompted by conventional postoperative imaging.

**Results:**

A total of 216 patients were included (median age 63 years), with 156 pelvic ring injuries and 60 acetabular fractures. Postoperative radiographs were obtained at a median of 3 days post-surgery. Changes in postoperative care based on radiographs occurred in 2 patients (3.3%) within the acetabular fracture group. Revision surgery triggered by conventional imaging was required in 1 patient (0.6%) among pelvic ring injuries. In all cases, additional CT imaging preceded definitive management changes.

**Conclusion:**

Routine immediate postoperative conventional radiography after surgical treatment of isolated pelvic fractures has minimal clinical impact. These findings support a shift toward selective, indication-based postoperative imaging, including targeted imaging in cases of poor intraoperative bone quality, anticipated noncompliance with protective mobilization protocols, particularly among geriatric patients, for whom the long-term consequences of radiation exposure may be of secondary importance, or limited intraoperative image quality.

## Introduction

The incidence of pelvic fractures is increasing, and these injuries represent a complex subset of musculoskeletal trauma associated with substantial morbidity, impaired functional outcomes, and significant healthcare utilization [1–3]. Their clinical relevance is highlighted by a bimodal distribution, as they are associated with high energy trauma and polytrauma in younger patients and low energy injury mechanisms in the geriatric population [4, 5]. The achievement and maintenance of anatomic reduction and stable fixation are pivotal determinants of long-term outcomes, as inadequate reduction has been correlated with adverse sequelae including pain, non-union, posttraumatic arthritis and secondary surgical interventions [6, 7].

Standard postoperative assessment of pelvic injuries traditionally relied on conventional radiological imaging, including anteroposterior (AP), inlet, and outlet views for pelvic ring fractures, or AP, obturator, and ala views for acetabular fractures, obtained shortly after surgery to verify fracture alignment and hardware placement [8]. However, the clinical utility of routine postoperative conventional radiography remains controversial, given the additional radiation exposure and healthcare costs. Studies in general and pelvic trauma have questioned whether plain radiographs significantly influence management decisions when intraoperative fluoroscopy and surgeon judgment already indicate satisfactory reduction and implant placement [9, 10].

In this study, we evaluated the clinical impact of postoperative X-rays during in-hospital management following surgical treatment of isolated pelvic fractures. Specifically, we assessed how these imaging studies influenced changes in treatment regimens and the need for revision surgery. No temporal restrictions were applied to the assessment of this impact throughout the primary in-hospital recovery phase. We hypothesized that the clinical impact of early postoperative X-rays in this patient population would be limited.

## Methods

### Study design and ethical considerations

This retrospective cohort study was approved by the Ethics Committee of the RWTH Aachen Faculty of Medicine (No. EK 24–113, internal registration CTC-A No. 24–108). Informed consent for data collection was obtained from all patients throughout their hospital treatment. The present study was performed in accordance with the Declaration of Helsinki in its most recent version as well as the Good Clinical Practice Guidelines, and is reported according to the Strengthening the Reporting of Observational Studies in Epidemiology (STROBE) guidelines [11].

### Study population

Patients aged ≥ 18 years who underwent surgical treatment for isolated pelvic ring injuries or acetabular fractures at an academic Level 1 trauma center in the interval of 2020 to 2023 were included. Eligible cases were identified through retrospective screening of administrative patient data using the 10th edition of the International Classification of Diseases (ICD). Patients were included if they received high quality intraoperative imaging, entailing at least two standard radiographic views that allowed for a comprehensive radiological assessment of the corresponding osteosynthesis. Radiographic views included AP/inlet/outlet projections for pelvic ring injuries and AP/obturator/ala projections for acetabular fractures. Postoperative X-rays were conducted on the same projections with at least two standard radiographic views. Furthermore, the evaluating radiologist must not have made any recommendations for further imaging in their written report due to limited assessability of the images. The adequacy of imaging was additionally independently evaluated by the senior authors of the article. Disagreements were solved by consensus.

Patients were excluded if they had concomitant injuries of the pelvis or other anatomical locations, pathological fractures or periprosthetic fractures. Patients were also excluded in the case of missing data, missing or inadequate intraoperative and postoperative imaging, or if postoperative CT imaging preceded conventional X-rays.

### Outcome measures and data collection

The primary outcome of this study was a change of standard operative procedures (SOP) based on the critical evaluation of postoperative x-rays. A change in patients` management was characterized as subsequent limitation of range of motion (ROM) or weight bearing, additional immobilization or a combination thereof due to the postoperative x-ray.

Secondary outcome was any implant- or fracture-related impediments that necessitated revision surgery, as indicated by the evaluation of postoperative X-rays. These complications included but were not limited to implant misplacement or loosening, secondary fracture dislocation, endoprosthetic joint dislocation, and peri-implant fracture.

The following data were also extracted from patient records: age, gender, American Society of Anesthesiologists (ASA) classification [12], substance abuse (nicotine, alcohol, drugs), psychiatric comorbidities, osteoporosis, dementia, “Arbeitsgemeinschaft für Osteosynthesefragen” (AO) fracture type and Judet/Letournel classification for acetabular fractures [13–15], type of definitive operative treatment, postoperative treatment regime, length of stay (LOS), time interval from definitive operative treatment to postoperative x-rays in days. The diagnosis of osteoporosis was considered present if it was explicitly documented in prior medical records, if bone mineral density assessment showed a T-score ≤ − 2.5, or if the patient was receiving osteoporosis-specific pharmacological therapy.

### Statistical analysis

Data was collected in a Microsoft Excel spreadsheet (Microsoft Corporation, Redmond, WA). Statistical analysis was performed using IBM SPSS Statistics for Windows (IBM Corp Version 29.0; Armonk, NY). The normality of continuous variables was assessed using Q-Q plots and the Shapiro-Wilk test. As all continuous variables were found to be non-normally distributed, continuous data were summarized using median and interquartile range (IQR). Categorical variables were presented as counts and percentages. Between-group comparisons were conducted using the Mann-Whitney U test for continuous variables and the chi-square test or Fisher Exact test for categorical variables, in cases of an expected frequency of 5 or less, for categorical variables. Statistical significance was defined as *P* < 0.05.

## Results

### Demographic characteristics

A total of 216 patients were included in this retrospective study, with a median age of 63 years (IQR 41–78) and a median ASA score of 2 (IQR 2–3). There was a balanced gender distribution throughout the study sample with 47.2% female patients. The incidence of osteoporosis was 19.9% with a preoperatively initiated osteoporotic treatment in 8.3% of the cases. Overall, there were 156 cases (72.2%) with pelvic ring injuries and 60 cases (27.8%) with acetabular fractures. AO type B injuries were the most common fracture type (Table 1) in both groups, while the associated both column fracture pattern was the most prevalent among included acetabular fractures (Table 2).

**Table 1:** Demographic characteristics of the study population

	Overall^§^	Pelvic ring injuries^§^	Acetabular fractures^§^	*P*-value*
N	216	156 (72.2%)	60 (27.8%)	-
Age (years)	63 (41–78)	64 (41–79)	61 (42–75)	0.73
Gender				
-Female	102 (47.2%)	82 (52.6%)	20 (33.3%)	**< 0.05**
ASA classification	2 (2–3)	2 (2–3)	2 (2–3)	0.69
Substance abuse				
-Nicotine -Alcohol -Drugs -Multiple substance abuse	58 (27%) 25 (12%) 9 (4%) 15 (7%)	41 (26.3%) 15 (9.6%) 6 (3.9%) 10 (6.4%)	17 (28.8%) 10 (16.6%) 3 (5%) 5 (8.8%)	0.68
Psychiatric comorbidity	32 (14.8%)	23 (14.7%)	9 (15%)	0.96
Dementia	30 (13.9%)	21 (13.5%)	9 (15%)	0.83
Osteoporosis -under treatment prior to admission	43 (19.9%) 18 (8.3%)	35 (22.4%) 14 (9%)	8 (13.3%) 4 (6.7%)	0.18
AO fracture type				
-A -B -C	20 (9.3%) 146 (67.6%) 50 (23.1%)	1 (0.6%) 121 (77.6%) 34 (21.8%)	19 (31.7%) 25 (41.7%) 16 (26.6%)	**< 0.05**

* Between-group comparisons were conducted using the Mann-Whitney U test for continuous variables and the chi-square test or Fisher Exact test, in cases of an expected frequency of 5 or less, for categorical variables. Statistical significance was defined as *P* < 0.05

§ Continuous data are presented as median values and interquartile range (IQR). Categorical variables are presented as counts and percentages

ASA: American Society of Anesthesiologists; AO: Arbeitsgemeinschaft für Osteosynthesefragen

**Table 2:** Judet/Letournel classification of included acetabular fractures [15]

	Acetabular fractures
N	60
Elementary fracture patterns	
-PW -PC -AW -AC -Transverse	6 (10%) 3 (5%) 1 (1.7%) 7 (11.7%) 6 (10%)
Associated fracture patterns	
-T-shaped -PC with PW -Transverse with PW -AC with PHT -ABC	5 (8.3%) 2 (3.3%) 5 (8.3%) 9 (15%) 16 (26.7%)

PW: posterior wall; PC: posterior column; AW: anterior wall; AC anterior column; PHT: posterior hemitransverse; ABC: associated both column

### Course of in-hospital treatment and principles of postoperative care

The median LOS was 10 days (IQR 8–15). The most commonly applied definitive operative treatment principle was closed reduction and internal fixation (CRIF) in pelvic ring injuries (*N* = 128, 82.1%), and open reduction and internal fixation (ORIF) in acetabular fractures (*N* = 48, 80%). Most patients were mobilized postoperatively with partial (toe-touch) weight bearing (*N* = 130, 60.2%), and in 5 cases (8.3%) with restrictions of hip motion (e.g., limitation of hip flexion > 90 degrees, avoidance of forced hip abduction/adduction/rotation). Postoperative X-rays were performed at a median time interval of 3 days (IQR 2–7) after definitive operative treatment (Table 3).

**Table 3:** In-hospital course of treatment and principles of postoperative care

	Overall^§^ *N* = 216	Pelvic ring injuries^§^ *N* = 156	Acetabular fractures^§^ *N* = 60	*P*-value*
LOS	10 (8–15)	10 (7–15)	12 (9–16)	0.06
Definitive operative treatment				
-ORIF -CRIF -Hip arthroplasty	76 (35.2%) 135 (62.5%) 5 (2.3%)	28 (17.9%) 128 (82.1%) 0	48 (80%) 7 (11.7%) 5 (8.3%)	**< 0.05**
Principles of postoperative aftercare				
-Weight-bearing				
Full Partial None -ROM restriction	54 (25%) 130 (60.2%) 32 (14.8%) 5 (2.3%)	54(34.6%) 73 (46.8%) 29 (18.6%) 0	0 57 (95%) 3 (5%) 5 (8.3%)	**< 0.05**

* Between-group comparisons were conducted using the Mann-Whitney U test for continuous variables and the chi-square test or Fisher Exact test, in cases of an expected frequency of 5 or less, for categorical variables. Statistical significance was defined as *P* < 0.05

§ Continuous data are presented as median values and interquartile range (IQR). Categorical variables are presented as counts and percentages

LOS: Length of stay; ORIF: Open reduction and internal fixation; CRIF: Closed reduction and internal fixation; ROM: Range of motion

### Outcome parameters

Following evaluation of the postoperative x-ray imaging, there was a change of SOP in two cases (3.3%), both within the subgroup of acetabular fractures with additional limitation of weight bearing. Both cases concerned osteoporotic geriatric patients with an anterior column posterior hemitransverse fracture according to the Letournel classification [15] and > 50% fragmentation of the quadrilateral surface, who were treated with ORIF with suprapectineal plate via an anterior intrapelvic approach. Following postoperative mobilisation, postoperative X-rays showed a mild acetabular incongruency as an indicator of acetabular medialisation tendency, which was not apparent intraoperatively after post-hoc evaluation of the corresponding imaging under knowledge of the pathology.

Operative revision based on the evaluation of conventional postoperative imaging was performed in one case (0.6%) in the subgroup of pelvic ring injuries, due to implant loosening with early secondary fracture dislocation. This case concerned a 78-year-old patient with a lateral compression injury of the pelvic ring (AO 61B2.1a), who was treated by percutaneous in situ fixation with a singular transsacral screw fixation in the S1 osseous corridor and was immediately mobilised postoperatively with full weight-bearing. Retrospective secondary evaluation of the intraoperative imaging confirmed adequate implant positioning and fracture reduction in adequate fluoroscopic projections.

Computed tomography imaging was performed prior to the formal indication of a change in postoperative care or revision surgery.

## Discussion

In this retrospective cohort study of 216 patients with isolated pelvic ring and acetabular fractures, early postoperative conventional X-rays had a very low clinical impact on subsequent management. Only 3.3% of patients with acetabular fractures experienced changes in standard postoperative treatment and 0.6% of patients with pelvic ring injuries underwent revision surgery based on findings from conventional X-rays. These findings support the hypothesis that routine early conventional postoperative imaging rarely changes clinical decisions when high-quality intraoperative fluoroscopy is available and carefully evaluated.

Our results are highly consistent with the most recent pelvic trauma–specific literature. A recent observational study evaluated the utility of routine postoperative radiographs in pelvic and acetabular trauma and demonstrated that early postoperative X-rays did not significantly impact postoperative management compared to intraoperative fluoroscopy, with very few postoperative care modifications attributable to postoperative imaging [9]. Importantly, radiological changes identified on postoperative radiographs in the referenced study were typically already evident on intraoperative imaging. In contrast, the postoperative radiological changes observed in our study were not detectable intraoperatively, even on retrospective secondary review of intraoperative images, and were largely attributed to the effects of remobilization and osteoporotic bone structure in geriatric patients. These findings reinforce the concept that early postoperative plain radiographs are largely redundant in centers with standardized intraoperative imaging protocols. Nevertheless, a residual, albeit small, risk of missing early radiological changes induced by postoperative mobilization remains if routine postoperative imaging is omitted, particularly in geriatric patients and those with osteoporotic bone structure.

Beyond pelvic trauma, a substantial body of orthopedic literature across multiple anatomical regions has consistently demonstrated the limited utility of routine early postoperative radiographs when intraoperative imaging guidance is used. Studies in hip fracture fixation have repeatedly shown that immediate postoperative x-rays rarely alter patient management, weight-bearing protocols, or the need for revision surgery [16–19]. Similar conclusions have been drawn in ankle fracture fixation [20, 21], spine surgery [22–24], shoulder, hip and knee arthroplasty [25–27], and ligament reconstruction [28]. Furthermore, a systematic review from Teo et al., which included studies on fractures of the hip, upper and lower extremities, came to the same result [10]. The consistency of these findings across subspecialties supports the broader principle that routine postoperative imaging is unlikely to influence clinical management in uncomplicated cases, provided that fixation has been adequately assessed intraoperatively and patient compliance with protective postoperative mobilization protocols is ensured.

Nevertheless, postoperative radiographs may serve not only for immediate verification of reduction quality and implant position, but also as a standardized baseline for later follow-up imaging. In routine clinical practice, radiographs obtained at 2–6 weeks postoperatively are frequently compared with early postoperative plain films to assess secondary displacement, implant migration, fracture healing, or loss of fixation under comparable projection conditions. In contrast, comparison of follow-up radiographs with intraoperative fluoroscopy or intraoperative CT/3D imaging may be limited by differences in patient positioning, image acquisition technique, magnification, and projection geometry [29]. Several authors have emphasized that serial conventional radiographs remain valuable during the later postoperative course, particularly when decisions regarding progression of weight bearing or confirmation of fracture union are required [10, 30, 31]. Phelps et al. demonstrated that postoperative radiographs were most clinically relevant during the interval between mobilization and confirmation of fracture healing rather than in the immediate postoperative setting [30]. Similarly, Sjølander et al. reported that scheduled follow-up radiographs after distal radius fixation occasionally identified secondary loss of reduction or implant-related complications requiring altered follow-up or revision surgery [31]. Therefore, although our findings question the necessity of routine immediate postoperative radiographs after satisfactory intraoperative imaging, a standardized baseline conventional radiograph may still offer practical value for longitudinal comparison during outpatient follow-up, particularly in patients at elevated risk for secondary displacement or fixation failure.

In contrast, cross-sectional imaging, particularly computed tomography, has demonstrated superior sensitivity for detecting clinically relevant findings after pelvic and acetabular fracture fixation. Several studies have shown that postoperative CT can identify residual displacement, implant malposition, and intra-articular hardware penetration not visible on conventional radiographs, occasionally leading to revision surgery [32–34]. As confirmation of this supporting evidence, CT imaging was subsequently performed prior to any formal indication for a change in postoperative aftercare or revision surgery in the population of the present study. However, even within this context, the routine use of postoperative CT remains controversial. Cronin et al. and Elnahal et al. both reported that although CT detected additional findings after pelvic ring fixation, only a small subset of these findings resulted in changes to management, raising questions regarding cost-effectiveness and radiation exposure in unselected patient populations [32, 33]. A recently emerging modality is intraoperative three-dimensional imaging, including computed tomography (CT), which enables immediate intraoperative assessment and correction at the cost of increased operative time and radiation exposure [35, 36]. Although hardware complexity and implementation costs have thus far limited widespread adoption of this modality, future research should focus on identifying specific patient populations that may benefit from its implementation.

The very low revision rate observed in the present study likely reflects the increasing reliance on meticulous intraoperative fluoroscopic control, which has been shown to correlate closely with postoperative radiographic findings. Previous work comparing intraoperative fluoroscopy with postoperative radiographs demonstrated near equivalence in detecting fixation-related issues, further supporting the notion that postoperative plain films seldom provide novel diagnostic information [29]. Given also the long known limitations of conventional radiography of pelvic trauma [37], it is consistent that postoperative x-rays in our cohort primarily confirmed intraoperative assessments rather than prompting intervention.

Performing a postoperative radiograph in an in-patient setting entail added radiation exposure, considerable effort and significant costs. While individual radiation doses from single X-rays are low, cumulative exposure from serial postoperative imaging can accrue and incrementally increase risk [38]. Further, modern radiation safety frameworks emphasize ALARA (As Low As Reasonably Achievable). Every imaging study should be justified by a clear expected change in patient management rather than performed routinely [39].Timely and straightforward imaging is primarily available on weekdays due to limited resources on weekends and public holidays. The patient must be transported from the ward to the radiology department using a transport service. During the patient’s waiting time, supervision must be ensured, particularly for geriatric, demented, or pediatric patients. Transferring the patient onto the radiographic table requires additional resources, especially in cases of increasing immobility. Following the examination, return transport to the patient’s room must be arranged. In addition to the costs associated with transport services, supervision during waiting periods, and the imaging itself including interpretation, the timing of the imaging must also be considered.

If these resources are unavailable on weekends or public holidays, discharge may need to be postponed until imaging can be performed. Unfortunately, relevant data on this aspect was not available in our study. Nevertheless, given rising healthcare costs, the necessity and implications of repeated imaging should be critically evaluated. Whether, in cases requiring revision surgery, earlier detection of findings during routine follow-ups at two or six weeks would have influenced patient outcomes, cannot be determined due to the limited number of cases. Finally, the issue of radiation protection must be critically evaluated. In particular, the pelvic region contains reproductive organs, which are highly sensitive to X-ray exposure. Gonadal shielding, such as the insertion of an additional lead plate during imaging, cannot always be ensured, as it may obscure essential components of fracture fixation. The revision cases identified in this study primarily involved geriatric patients who demonstrated limited adherence to protective postoperative mobilization protocols due to underlying neurological conditions. In these isolated cases, radiological changes occurring after mobilization may not have been detected if routine postoperative imaging had been omitted. However, these observations should be interpreted with considerable caution, as they were confined to a very small subgroup of the overall study population. In younger patients, who can be assessed and re-evaluated clinically with high reliability, and for whom the long-term consequences of radiation exposure are more significant, the necessity of postoperative radiographs must be carefully reconsidered.

### Strengths and limitations

A major strength of this study is the inclusion of a homogeneous cohort of patients with isolated pelvic ring or acetabular fractures, minimizing confounding from associated injuries that may independently influence postoperative management. Furthermore, the absence of a predefined temporal restriction allowed assessment of the clinical impact of postoperative radiographs throughout the entire in-hospital treatment course, rather than within an arbitrarily short observation window. The requirement for high-quality intraoperative and postoperative imaging further strengthens the validity of the findings.

Nevertheless, several limitations must be acknowledged. The retrospective single-center design limits generalizability and may reflect institution-specific surgical expertise and imaging standards. Further, inherent limitations of retrospectivity such as treatment, observation and interpretation bias must be acknowledged. Routine postoperative CT imaging was not performed, potentially underestimating subtle fixation-related abnormalities detectable only with cross-sectional imaging. Finally, although routine postoperative x-rays demonstrated minimal impact in this cohort, their utility in selected high-risk scenarios cannot be excluded.

## Conclusion

In patients with isolated pelvic ring or acetabular fractures treated surgically under established intraoperative imaging control, routine immediate postoperative X-rays appear to have minimal clinical impact. Changes in postoperative management or the need for revision surgery triggered by these images were exceedingly rare. These findings support a shift toward selective, indication-based postoperative imaging strategies, including targeted postoperative imaging in cases of intraoperatively identified poor bone quality, anticipated noncompliance with protective postoperative mobilization protocols, particularly among geriatric patients, for whom the long-term consequences of radiation exposure may be of secondary importance, or when intraoperative image quality is limited. In selected situations, alternative strategies, such as performing computed tomography upfront instead of routine postoperative X-rays, or incorporating intraoperative three-dimensional imaging to enable immediate assessment and correction in complex fracture patterns and dysmorphic osseous anatomy, may further optimize imaging utilization. Adoption of such patient-tailored approaches has the potential to reduce radiation exposure, healthcare costs, and resource utilization without compromising patient safety or quality of care.

## Data Availability

The data collected will be stored securely at our institute for 10 years. During this period, they are still available upon request. After 10 years, the data will be deleted, however, all the datasets analyzed or generated during this study will be available from the corresponding author upon reasonable request.
